# Effects of a Food-Shaping Agent on the Texture and Palatability of Hospital-Pureed Meat: A Comparison of Subjective and Instrumental Assessments

**DOI:** 10.3390/foods14203574

**Published:** 2025-10-21

**Authors:** Ya-Ting Kuo, Pey-Rong Chen, Suh-Ching Yang

**Affiliations:** 1 School of Nutrition and Health Sciences, Taipei Medical University, Taipei 11031, Taiwan; yatingkuo@ntuh.gov.tw; 2 Department of Dietetics, National Taiwan University Hospital, Taipei 100225, Taiwan; prchen@ntuh.gov.tw; 3 Research Center of Geriatric Nutrition, College of Nutrition, Taipei Medical University, Taipei 11031, Taiwan; 4 Nutrition Research Center, Taipei Medical University Hospital, Taipei 11031, Taiwan; 5 School of Gerontology and Long-Term Care, College of Nursing, Taipei Medical University, Taipei 11031, Taiwan

**Keywords:** dysphagia, texture-modified diet, pureed food, Texture Profile Analysis (TPA), international dysphagia diet standardisation initiative (IDDSI), Universal Design Foods (UDF)

## Abstract

(1) Background: This study compared subjective and objective texture classifications of hospital-provided pureed meat dishes and evaluated the impact of adding a food-shaping agent on the consistency of the food. (2) Methods: In total, 18 common pureed meat dishes (pork, chicken, and fish) from a medical center were tested. Subjective classification was conducted according to the International Dysphagia Diet Standardisation Initiative (IDDSI) level 4 criteria, and an objective texture analysis was performed using a Texture Profile Analysis (TPA), with hardness values interpreted via the Universal Design Foods (UDF) framework. (3) Results: Only six of the 18 dishes (33%) met all IDDSI level 4 tests in their original form, despite visually resembling purees. After the addition of 1% of a food-shaping agent, all samples passed IDDSI criteria, indicating enhanced textural consistency and a reduced risk of swallowing complications. TPA data confirmed that all samples, both with and without the food-shaping agent, met UDF stage 4 hardness standards (<5 × 10^3^ N/m^2^), ensuring appropriate structural integrity for safe swallowing. The addition of food-shaping agents significantly increased the hardness and adhesiveness (*p* < 0.001), while the cohesiveness remained unchanged. (4) Conclusions: These findings highlight discrepancies between visual/subjective assessments and objective measurements and support the use of combined IDDSI- and TPA-based verification to improve dietary safety and reproducibility in dysphagia care.

## 1. Introduction

Dysphagia has emerged as a critical health concern in the context of global aging populations. It is estimated that approximately 8–16% of the general population experiences some degree of swallowing difficulty, with a significantly higher prevalence of 27–30% among individuals aged 65 years and older [1,2]. According to projections from Taiwan’s Ministry of the Interior, the country will become a super-aged society by 2025, with over 20% of the population aged 65 years or older [3], underscoring an urgent need for enhanced dysphagia care.

Texture-modified diets (TMDs) remain one of the primary nutritional interventions for dysphagia management, helping to reduce the risks of choking and aspiration pneumonia [4]. In clinical and long-term care facilities, TMDs are typically prepared by kitchen staff using blenders or grinders to process regular meals into minced or pureed forms, thereby alleviating the chewing and swallowing burden for older adults [5]. However, these preparation processes often lack standardized protocols and are rarely guided by objective indicators of textural safety, potentially increasing associated risks [6].

To enhance the safety and consistency of dysphagia diets, standardized texture classification systems have gradually been promoted worldwide. Among them, the International Dysphagia Diet Standardisation Initiative (IDDSI) has become the most widely adopted clinical framework. The IDDSI system utilizes simple tools such as spoon tilt and fork drip tests to classify food textures [7]. While it offers ease of use and dissemination, its results heavily rely on the assessor’s subjective judgment and are susceptible to variations in sample appearance, evaluator skill, and sensory perceptions.

By comparison, the Universal Design Foods (UDF) framework proposed by the Ministry of Health, Labour and Welfare in Japan offers an objective classification system based primarily on the numerical hardness of foods, supplemented by indicators such as viscosity and form. This enables more consistent and reproducible food design [8]. However, in most clinical settings, the application of UDF remains limited, and texture assessments still predominantly rely on the IDDSI framework.

Although the IDDSI framework has been widely adopted in clinical practice worldwide, its assessment methods tend to rely on the evaluator’s immediate judgment of food properties and the force applied, leading to a certain degree of subjectivity and personal bias. To improve the consistency and objectivity of texture classification, clinical practice urgently requires the integration of instrumental analyses as complementary quantitative support. The Texture Profile Analysis (TPA), for instance, simulates various chewing scenarios through different probe types and pressure settings, generating representative quantitative parameters [9]. These data contribute to the standardization of food design and quality control, thereby facilitating implementation of personalized and safe dietary interventions in clinical situations.

In dysphagia rehabilitation, dietary management plays a vital supportive role. The accuracy and consistency of a food’s texture directly impact the safety of oral intake and the effectiveness of recovery [10]. Misclassification of texture may lead to choking, residues, or even aspiration pneumonia, making reliability in texture classification crucial for both training and care processes [11]. In this study, we aimed to establish a dual-modality texture grading model, combining subjective and objective evaluations, to enhance dietary safety and reproducibility in dysphagia care. Therefore, this study focused on pureed meat dishes from a hospital in northern Taiwan to address the limitations of IDDSI’s subjectivity and enhance food preparation safety and visual consistency. A commercial food-shaping agent was applied to improve the visual appeal and textural stability. Such shaping agents are commonly polysaccharide-based, providing water-binding and gel-forming properties that enhance cohesion, shape retention, and surface adhesiveness of pureed meals, thereby improving their acceptability in clinical practice. Textures were graded using both subjective IDDSI tests and objective TPA. Hardness values obtained from the TPA were interpreted using the UDF classification to assess consistency between the grading methods. In Taiwan, the IDDSI framework has been widely adopted in hospital kitchens, while the UDF classification is rarely applied. Comparing both systems in this context allows us to identify discrepancies between subjective and objective assessments and highlight the need for combined use to ensure dietary safety.

## 2. Materials and Methods

### 2.1. Sample Selection and Preparation

Food samples used in this study were collected from texture-modified meals served to inpatients at a medical center in northern Taiwan. These dishes were chosen from the standard inpatient menu of a large medical center. While they may not represent all hospitals nationwide, pork, chicken, and fish are the most commonly prepared protein dishes in Taiwanese clinical settings. To ensure representation of different meat types and preparation methods, 18 pureed meat dishes in total were selected—six from each meat category. Pork samples included dishes made with ground hind leg and pork shoulder; chicken samples included sliced chicken breast and boneless chicken thigh; and fish samples comprised dishes using white catfish and boneless tilapia.

**Figure 1:**
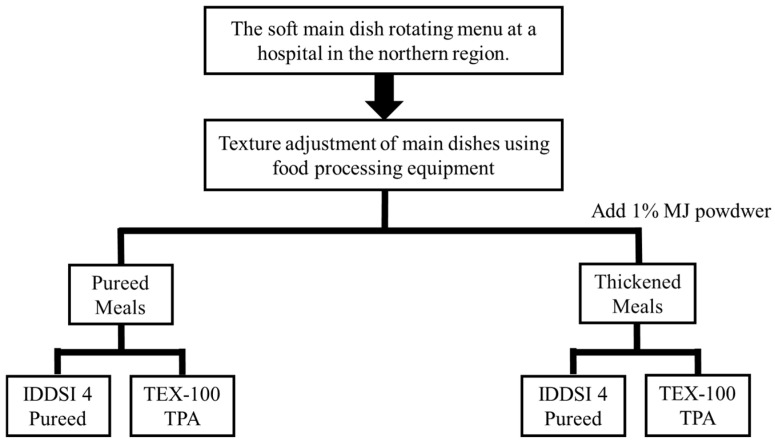
Workflow of sample processing and experimental procedures in this study. IDDSI, International Dysphagia Diet Standardisation Initiative; TPA, Texture Profile Analysis; TEX-100, Commercial texture analyzer model used for instrumental testing; MJ, mousse and jelly shaping powder used to modify the texture of meals.

All samples were prepared by institutional kitchen staff following routine cooking procedures and immediately pureed using a commercial food processor. The purees were divided into two treatment groups: one without the addition of a food-shaping agent, representing standard pureed meals for individuals with impaired mastication, and the other with an added commercial food-shaping agent (mousse and jelly powder, FoodCare Co., Ltd., Kanagawa, Japan), applied at 1% *w*/*w*, to improve the visual appearance and enhance the textural stability for patients requiring IDDSI level 4 (pureed) texture diets. The shaping agent was supplied in powder form and incorporated immediately after pureeing, without additional heating. The 1% *w*/*w* level was selected according to the manufacturer’s recommended dosage, which is widely applied for puree shaping. This concentration has also been incorporated into the hospital kitchen’s routine standard operating procedure (SOP) for preparing texture-modified meals. Upon preparation, all samples were immediately analyzed at a temperature of 28 ± 2 °C and were not refrigerated. Detailed sample processing and the experimental workflow are illustrated in Figure 1. The shaping agent contains polysaccharide-based ingredients such as dextrin, xanthan gum, glucomannan, and tara gum, according to the manufacturer. The same proportion was used across all dishes to ensure consistency.

### 2.2. Subjective Texture Classification: IDDSI Testing

Subjective texture grading was performed in accordance with level 4 (pureed) food criteria outlined in version 2.0 of the IDDSI framework [7]. To minimize inter-rater variability, all assessments were conducted by a single trained clinical dietitian with more than 10 years of experience in dysphagia nutrition care. Each sample was tested three times, and a sample was considered noncompliant if it failed to meet level 4 criteria in any of the three trials.

Standardized utensils were used throughout the testing process, including a fork with a tine width of 1.5 cm and spacing of 0.4 cm, as well as a standard spoon. Three IDDSI-recommended tests were conducted to evaluate the sample texture. The fork pressure test involved pressing the bottom of the fork onto a sample to determine whether it retained distinct indentation marks, indicating sufficient structural strength and cohesiveness. The fork drip test assessed whether the sample could maintain its shape when placed on the fork; while a small amount of food was permitted to droop through the fork slots and form a tail, continuous dripping or visible liquid flow resulted in a failed classification. Lastly, the spoon tilt test examined the sample’s ability to slide off a tilted spoon in a single cohesive mass without breaking apart or leaving excessive residue. Only a minimal amount of food remaining on the spoon’s surface was considered acceptable, reflecting appropriate levels of adhesiveness and cohesiveness.

### 2.3. UDF-Based Texture Classification

Hardness values obtained from the TPA were classified based on the UDF framework established by the Japan Nursing Food Council. This system categorizes food textures into four stages (stages 1 to 4), using predefined hardness thresholds and swallowing-related indicators [8]. These classification criteria and their corresponding hardness limits are summarized in Table 1. In this study, viscosity measurements were not conducted; therefore, preliminary classification was performed solely based on measured hardness values.

### 2.4. Objective Texture Measurement: Texture Profile Analysis (TPA)

An objective texture analysis was performed using the TPA method with a TEX-100N texture analyzer (Kyowa Interface Science, Niiza—City, Saitama, Japan). Each sample was placed into a cylindrical polyacetal test container (40 mm in diameter, 20 mm in height, and 15 mm in depth) with a target sample weight of 18 ± 1 g. Surface testing was conducted at 28 ± 2 °C. Compression testing was carried out using a flat cylindrical probe with a diameter of 20 mm, set to a compression ratio of 66.67% and a speed of 1.0 mm/s. Each sample was tested six times; the highest and lowest values were excluded, and the mean of the remaining four measurements was used for statistical analysis.

Three key texture parameters were extracted from the TPA results. Hardness, expressed in newtons per square meter (N/m^2^), equivalent to pascal (Pa), was defined as the maximum force recorded during the first compression cycle, reflecting the sample’s resistance to deformation and serving as an indicator of initial masticatory force. Adhesiveness, measured in joules per cubic meter (J/m^3^), represented the negative area required to detach the probe from the sample, indicating the tendency of the food to adhere to oral surfaces such as the tongue or pharyngeal walls, and was associated with post-swallowing residue. Cohesiveness, a unitless ratio calculated by dividing the force during the second compression by the first, represented the internal structural integrity of the food and its ability to maintain form during mastication. Note: 1 N/m^2^ = 1 Pascal (Pa), following SI unit conventions.

Texture parameters were summarized using Excel 365 and analyzed with SPSS (vers. 29; IBM, Armonk, New York, NY, USA). Hardness values were classified based on UDF criteria (Table 1) and compared to IDDSI-based subjective evaluations to assess the classification consistency (Table 2). Statistical methods are detailed in the following section.

### 2.5. Statistical Analysis

Statistical analyses were performed using Excel 365 and SPSS (vers. 29; IBM Corp., Armonk, NY, USA). Continuous variables are presented as the mean ± standard deviation (SD). Data normality was evaluated using the Shapiro–Wilk test. For normally distributed variables, paired comparisons before and after food shaping-agent addition were conducted using a paired Student’s *t*-test. For non-normally distributed paired data, the Wilcoxon signed-rank test was applied, as it is more appropriate for within-subject comparisons than the Mann–Whitney U test, which is intended for independent samples. To compare texture parameters across different meat types (pork, chicken, and fish), the Kruskal–Wallis test was employed, followed by Dunn’s test for post hoc multiple comparisons. All statistical tests were two-tailed, and *p* < 0.05 was considered statistically significant.

## 3. Results

### 3.1. Evaluation of Pureed Meat Dishes

In total, 18 pureed meat-based main dishes were subjected to IDDSI level 4 (pureed) subjective testing, which included a fork pressure test, fork drip test, and spoon tilt test. The evaluation criteria required that samples demonstrate appropriate cohesiveness and adhesiveness—retaining structural integrity under fork pressure, maintaining mound formation without continuous dripping on a fork, and sliding off a tilted spoon in one cohesive mass without fragmentation or excessive residue.

Under the condition without a food-shaping agent, only six of the 18 samples (33%) successfully met all IDDSI level 4 requirements. These compliant samples were derived from dishes prepared using ground pork, chicken breast, and white catfish. Among the pork-based items, two of the three dishes made with ground hind leg passed all tests, whereas the braised minced pork with pickled cucumber failed due to excessive fluidity that interfered with fork indentation and cohesive spoon release. In contrast, none of the pork shoulder cube dishes met the criteria, suggesting that this meat type may produce harder or more adhesive textures unless modified.

All three boneless chicken thigh dishes failed IDDSI tests. Specifically, the red yeast and teriyaki preparations left substantial residue on the spoon during tilt testing, indicating high adhesiveness, while the pepper-salt version lacked adequate form and spread too easily. For chicken breast slices, two out of three samples passed, with only the version containing papaya failing due to excess moisture. In the fish category, two of the white catfish dishes passed IDDSI testing, while the stir-fried diced catfish with bell peppers failed as a result of excessive fluidity. All three boneless tilapia dishes failed both fork and spoon tests, reflecting insufficient cohesiveness and weak structural integrity in the absence of a shaping agent.

Overall, although these hospital-prepared dishes visually resembled pureed foods, a substantial portion did not meet the structural requirements of IDDSI level 4 without texture modification. To further validate these findings, the hardness values of all samples were measured using the TPA and interpreted using the UDF stage 4 criteria, which define the upper hardness limit as 5 × 10^3^ N/m^2^. All samples, regardless of compliance with IDDSI, were found to fall within the UDF-defined hardness range. This indicates that from a structural integrity standpoint, the samples were technically safe for swallowing without the need for chewing, despite inconsistencies in their subjective IDDSI performances. Results are summarized as shown in Table 3, Table 4 and Table 5.

### 3.2. Evaluation of Pureed Dishes After Addition of a Food-Shaping Agent

After the addition of 1% of a mousse and jelly shaping powder (FoodCare Co., Ltd., Kanagawa, Japan), all 18 pureed meat dishes were retested using the three IDDSI level 4 subjective assessments. All samples passed the fork pressure, fork drip, and spoon tilt tests, meeting the full criteria for IDDSI level 4. This outcome indicates that the incorporation of the food-shaping agent effectively improved the textural stability and performance in subjective testing. Notably, all 12 samples that had previously failed were upgraded to a compliant status after adding the food-shaping agent, raising the overall IDDSI compliance rate from 33% to 100%. In addition, hardness values obtained via the TPA demonstrated that all samples still fell within the UDF stage 4 safety threshold (hardness < 5 × 10^3^ N/m^2^) following food-shaping agent treatment. This suggests that the improved structural integrity remained within the range considered safe for swallowing (Table 6).

### 3.3. Texture Parameter Differences Before and After Addition of a Food-Shaping Agent

To further examine the effect of the food-shaping agent, texture parameters measured by the TPA were compared before and after treatment across all 18 samples (*n* = 18) (Table 7). Results showed a significant increase in hardness, rising from 231.97 ± 206.66 to 699.36 ± 261.48 N/m^2^ (*p* < 0.001), indicating a marked enhancement in the structural strength. Adhesiveness significantly increased from 38.74 ± 56.1 to 139.21 ± 56.94 J/m^3^ after addition of the food-shaping agent (Wilcoxon signed-rank test, *p* < 0.001), indicating a marked rise in the adhesive strength. The relatively large SD values mainly reflect the intrinsic variability among the 18 different hospital-prepared dishes, which differed in meat type, cooking method, and moisture distribution. Despite this variability, consistent trends were observed across all dishes, and the paired statistical comparisons still yielded highly significant differences, supporting the reliability of the findings. In contrast, cohesiveness showed a slight reduction from 0.88 ± 0.06 to 0.78 ± 0.06, but the change was not statistically significant (*p* = 0.200). These results indicated that the food-shaping agent had a substantial effect on increasing both the hardness and adhesiveness, which may help improve bolus formation and intraoral stability prior to swallowing. However, its effect on the internal structural cohesion, as reflected by cohesiveness, appeared to be relatively limited.

### 3.4. Texture Differences Among Meat Types Before and After Addition of the Food-Shaping Agent

This study further examined the effects of the food-shaping agent on different types of meat—pork, fish, and chicken—by comparing their TPA-derived texture parameters (hardness, cohesiveness, and adhesiveness) before and after treatment (Table 8). Before the addition of the food-shaping agent, chicken samples exhibited significantly higher hardness (354.84 ± 301.21 N/m^2^) compared to pork (185.16 ± 78.43 N/m^2^) and fish (155.91 ± 90.42 N/m^2^) (*p* < 0.05). Adhesiveness was also notably higher in chicken samples (73.56 ± 79.90 J/m^3^). No significant differences in cohesiveness were observed among the three meat types. Following the addition of the food-shaping agent, all three types showed substantial increases in hardness. Chicken and fish samples remained significantly harder than pork samples (*p* < 0.05), with chicken reaching the highest hardness value of 872.43 ± 302.88 N/m^2^. In terms of adhesiveness, both fish and chicken samples had significantly higher values than pork (149.78 ± 33.32 and 168.73 ± 64.15 J/m^3^, respectively), and the differences were statistically significant. Cohesiveness exhibited a general downward trend across all groups, but the differences among meat types were not statistically significant.

## 4. Discussion

### 4.1. Conventional Pureed Dishes vs. IDDSI Level 4 Criteria

This study revealed that under conditions without a food-shaping agent, only six of 18 samples (33%) met all three subjective test criteria for IDDSI level 4. The lowest compliance rates were observed in dishes prepared with boneless chicken thigh and snapper (Table 3, Table 4 and Table 5). Although all samples visually appeared to be puree-like, many failed to achieve the expected criteria—such as mound formation, non-dripping behavior, and cohesive spoon release—due to excessive fluidity or adhesiveness during the fork pressure, fork drip, and spoon tilt tests [7]. These results highlight that clinically prepared pureed meals, despite their soft appearance, may still pose swallowing risks due to unstable texture properties. Subjective perception alone is often insufficient to ensure safety, and inconsistent classification may lead to clinical miscommunication or inappropriate dietary recommendations [13]. Therefore, it is recommended that food preparation workflows incorporate routine and standardized texture testing mechanisms. Where necessary, the use of thickeners or food-shaping agents should be considered to adjust physical properties and enhance dietary safety for patients with dysphagia.

### 4.2. Effects of the Food-Shaping Agent on IDDSI Level 4 Compliance

Common polysaccharide-based ingredients found in food-shaping agents—such as dextrin, glucomannan, xanthan gum, and tara gum can improve textural properties such as hardness, chewiness, springiness, and cohesiveness [14,15,16,17]. This improvement is mainly due to their water-binding capacity, which reduces cooking loss and enhances juiciness; their ability to form gel networks that reinforce meat protein structure; their role in stabilizing fat-water emulsions; and their interactions with meat proteins, which influence gel strength and elasticity [18]. However, the magnitude of these effects varies depending on the type of meat product, the specific dietary fiber used, and its concentration [18]. In Taiwan, food-shaping powders have gradually been adopted in hospital kitchens, particularly for patients requiring long-term pureed meals, although they are not yet standard practice in all institutions. After the addition of 1% of mousse and jelly shaping powder, all 18 samples successfully passed all three IDDSI level 4 subjective tests, raising the overall compliance rate from 33% to 100% (Table 6). Furthermore, all hardness values measured by the TPA remained within the safety threshold defined for UDF stage 4 (<5 × 10^3^ N/m^2^), indicating that the improved structure did not result in excessive hardness and remained safe for swallowing. These findings confirmed that food-shaping agent incorporation effectively enhanced the structural stability, shape retention, and visual uniformity of these pureed foods, while also addressing IDDSI failures caused by high fluidity or poor consistency [19]. In this study, shaped samples demonstrated improved mound formation and reduced fluidity in both the spoon tilt and fork drip tests. Spoon residue was also significantly reduced, indicating that shaping helped mitigate texture instability caused by variable moisture levels and excessive adhesiveness.

On the other hand, the effectiveness of food-shaping agents differs among meat types due to compositional and structural variations due to the differences in water-holding capacity, pH, and protein denaturation further explain the variability in textural outcomes [20]. For example, chicken, with high protein density and low fat, tends to form firmer textures, whereas pork’s higher fat and connective tissue content enhances juiciness and cohesiveness. Moreover, fish, with delicate fibers and higher water content, responds mainly through water retention and gel formation [21].

In addition, applying to a food-shaping agent may enhance food presentation and patient acceptance, making texture-modified diets in clinical settings not only safer but also more reproducible [22]. Beyond compliance with IDDSI and UDF standards, shaping agents may also influence swallowing ease, patient satisfaction, and nutritional intake. Although our study did not directly measure these outcomes, shaping agents are considered in clinical practice to improve mealtime experience and acceptance. Future studies can therefore include direct assessments of swallowing function, patient-reported outcomes, and nutrient bioavailability. The shaping strategy proposed in this study may serve as a reference for developing standardized operating procedures (SOPs) in institutional food preparation, ultimately improving the mealtime experience and quality of life for individuals with dysphagia.

### 4.3. Major Effects of the Food-Shaping Agent on Textural Properties

TPA data indicated that the addition of a food-shaping agent significantly increased both the hardness and adhesiveness of the pureed dishes—from 231.97 to 699.36 N/m^2^, and from 38.74 to 139.21 J/m^3^, respectively—with statistically significant differences (*p* < 0.001). In contrast, cohesiveness decreased slightly from 0.88 to 0.78, but the change was not statistically significant (Table 7). These results align with previous studies demonstrating that polysaccharides can enhance the consistency and stability of food products [23]. The observed increase in adhesiveness may serve as an indicator of improved textural integrity, helping foods retain their shape, reduce dispersion in the oral cavity, and enhance bolus stability prior to swallowing [24]. In this study, the relatively large SD values observed in hardness and adhesiveness mainly reflected dish-to-dish variability, as the 18 hospital-prepared dishes differed in meat type, cooking method, and moisture distribution. Although the overall trends and statistical significance remained robust, this heterogeneity should be acknowledged as a limitation. Future studies with larger sample sizes and multiple shaping agent concentrations are encouraged to apply paired analyses or two-way ANOVA to better account for dish-level variability and to provide more detailed insights into the interaction between shaping agents and different food matrices. However, excessive adhesiveness may also increase post-swallowing residue and potentially elevate the risk of coughing or aspiration [25]. Therefore, clinical evaluation should be complemented by IDDSI’s spoon tilt test to assess bolus release and the oral residue performance. In our study, adhesiveness increased after shaping, yet spoon tilt test results improved, suggesting that adhesiveness at this level enhanced bolus stability without producing excessive residue. This finding highlights the dual role of adhesiveness and suggests that moderate increases may be beneficial, whereas excessive levels could still pose risks in clinical practice.

However, while the TPA data show that hardness and adhesiveness increase significantly with the shaping agent in this study, the literature indicates that not all fiber/binder additions lead to increases in these texture parameters. For example, the study on restructured spent hen meat with high β-glucan (barley, oat) found lower hardness, gumminess, chewiness compared to control [26]. Likewise, bacterial cellulose in tilapia sausages altered adhesiveness but did not uniformly increase it in all fat/fiber combinations [27]. These differences emphasize that the specific type, amount, and interaction of non-meat components with protein/fat matrix are critical in determining whether hardness/adhesiveness/cohesiveness increases, remains stable, or even decreases.

It is worth noting that although cohesiveness did not significantly change in numerical terms, improvements were observed in the IDDSI assessments. Shaped samples demonstrated better mound formation and lower drip tendency in the fork drip test, suggesting enhanced internal cohesion. Additionally, while adhesiveness significantly increased, it paradoxically improved the spoon test performance by promoting cohesive bolus release and reducing surface residue. This finding contrasts somewhat with that of Wong et al. (2023), who reported a strong correlation between adhesiveness and stickiness (*p* < 0.0001), noting that high-adhesive samples often exhibited greater cohesion and more residue on the spoon [28]. The differing results in our study may be attributed to the specific characteristics of pureed meat products, including their moisture distribution, colloidal structure, and emulsified fat content. As demonstrated by Wu et al. (2009), the interaction between myofibrillar proteins and emulsified fats forms a gel-like network, and its texture greatly depends on how moisture and fat are distributed within the product [29]. These structural factors can directly influence instrumental texture attributes such as hardness, cohesiveness, and adhesiveness.

### 4.4. Complementarity and Limitations of IDDSI and Instrumental Analysis

The IDDSI provides a globally recognized framework for classifying food textures. It uses simple methods such as the spoon tilt and fork drip tests to simulate eating scenarios across different levels of chewing and swallowing abilities. This approach offers advantages such as ease of use, rapid assessment, and low cost, making it particularly suitable for hospitals and long-term care facilities [30]. However, the results remain highly dependent on the evaluator’s experience and technique and may be influenced by factors such as the sample shape, applied force, and visual interpretation, potentially leading to inconsistent classification and unreliable risk assessments [28]. Studies noted that relying solely on IDDSI testing may overlook subtle yet clinically important differences in food texture and may inadequately reflect the actual swallowing behavior of samples with high viscosity or adhesiveness [31].

In contrast, the TPA is an instrumental method that quantifies multiple texture-related parameters such as hardness, adhesiveness, and cohesiveness. It provides reproducible and objective data that are essential for evaluating food texture and simulating oral processing [32]. To better reflect the deformation patterns that occur during mastication, particularly in soft-textured meats, the current study employed a flat, 20-mm cylindrical probe with double compression testing. This configuration was designed to more realistically simulate oral processing and capture texture parameters relevant to clinical swallowing safety. Previous instrumental studies supported this methodology. Wee et al. (2018) demonstrated that texture properties derived from the TPA correlated with actual eating behaviors, including bite size, chewing rate, and oral exposure time [33]. Rahman et al. (2021) further emphasized the importance of standardized probe geometry and compression settings across food types to ensure objective and reproducible TPA outcomes [34]. Additionally, research on chewing and swallowing patterns in healthy individuals showed that different food textures elicit distinct oral processing strategies, underscoring the importance of TPA configurations that reflect physiological conditions [35]. Collectively, these findings support the TPA as a reliable and reproducible method for assessing food texture. It enhances the accuracy of safety evaluations and contributes to the standardization of product development and quality control in both clinical and industrial settings.

While both the IDDSI framework and TPA offer valuable approaches for evaluating food textures, findings from this study revealed notable discrepancies between the two methods. Most samples satisfied the hardness threshold defined by UDF stage 4, yet several failed to meet the IDDSI level 4 criteria due to excessive adhesiveness or high fluidity. This inconsistency highlights the limitations of exclusively relying on either subjective or instrumental evaluations. Specifically, the TPA provided quantifiable data on hardness, cohesiveness, and adhesiveness, but it could not fully capture the nuanced sensory attributes observed during clinical testing. Di Monaco et al. (2008) found that instrumental measurements closely reflected hardness and springiness but showed weaker alignment with sensory cohesiveness ratings [30]. These findings suggest that neither method alone is sufficient to comprehensively evaluate the safety and suitability of texture-modified diets.

In summary, these findings illustrate the distinct yet synergistic advantages of the IDDSI and TPA. The IDDSI remains a practical tool for rapid clinical screening, whereas the TPA provides quantifiable data that can inform food design and quality control. To enhance the accuracy, safety, and standardization of texture-modified diets, a dual-assessment framework is recommended using IDDSI as a clinical foundation, supplemented by objective measurements when further verification is warranted.

### 4.5. Strengths and Limitations

This study utilized hospital-prepared meals and incorporated both subjective evaluation by IDDSI-based testing and objective evaluation by the TPA, establishing an assessment model that integrates subjective and objective approaches for texture evaluation. By including a variety of commonly consumed meat types and preparation methods, the study closely reflects typical clinical settings. Photographic documentation combined with side-by-side classification enabled direct comparisons between conventional pureed textures and those conforming to IDDSI level 4 standards. The overall design supports greater efficiency and standardization in evaluating texture-modified diets, laying the groundwork for the development of SOPs in clinical nutrition care.

However, this study has several limitations. First, the representativeness of the selected 18 dishes may be limited, as they were drawn from a single institution. Future studies should expand to include other food types and multiple institutions to enhance generalizability. Although all samples were prepared using a standardized protocol, potential variability in marination time, heating conditions, and handling procedures may have introduced minor differences in texture performance between dishes. Second, only one evaluator performed the IDDSI tests. Although this approach helped reduce inter-rater variability, future studies should involve multiple trained evaluators to further enhance reliability. Third, the analysis focused on three key texture parameters relevant to dysphagia management: hardness, cohesiveness, and adhesiveness. These attributes were objectively measured using the TPA. However, viscosity was not assessed, since the UDF classification system is primarily based on hardness values. The absence of viscosity testing may have limited the accuracy of the safety evaluations, especially for samples with higher fluid contents. Future studies are encouraged to incorporate simple viscosity assessment tools, such as the IDDSI Flow Test or rheometer-based techniques, to generate a more-comprehensive texture profile. Integrating these methods could enhance the precision and clinical relevance of safety evaluations for texture-modified diets.

## 5. Conclusions

This study evaluated commonly used hospital pureed meat dishes using both subjective IDDSI testing and an instrumental TPA, comparing samples with and those without addition of a food-shaping agent and classifying them according to UDF standards. Results showed that only approximately 30% of unshaped samples met IDDSI level 4 criteria, primarily due to excessive fluidity or adhesiveness. After the addition of 1% of a food-shaping agent, all samples passed both subjective and objective assessments, and all hardness values remained within the UDF stage 4 safety range. This confirmed the food-shaping agent’s effectiveness in improving the structural stability and swallowing safety of the pureed foods.

Additionally, this study demonstrated discrepancies between subjective IDDSI grading and objective TPA measurements, underscoring the potential limitations of relying on a single assessment method, which may underestimate risks in certain foods. It is therefore recommended that clinical evaluations and preparation of texture-modified diets adopt an integrated approach that combines both subjective and objective assessments to improve reproducibility and safety. The shaping procedure and analytical model proposed in this study can serve as a reference for SOP development and product formulation in clinical and foodservice settings, ultimately enhancing the nutritional care and mealtime experience of individuals with dysphagia.

## Figures and Tables

**Table 1:** Classification criteria and hardness ranges of UDF [8].

Classification	Chewing Ability	Swallowing Characteristic	Hardness Upper Limit (N/m^2^)	Physical Properties
Stage 1 Easy to chew	Cannot chew hard solids	Swallowing is normal	<5 × 10^5^	May have shape; if so, must be soft and breakable with the tongue
Stage 2 Can swallow without chewing	Cannot chew hard solids	Swallowing semi-liquid foods is possible	<5 × 10^4^	May have shape; if so, must be soft enough to break with the tongue
Stage 3 Breakable with the tongue	Very limited chewing ability	Difficulty swallowing slightly solid foods	Flow type: <10^4^ Gel type: <2 × 10^4^	Should not retain shape; considered non-solid food with a homogeneous texture
Stage 4 No chewing required	Cannot consume solid foods	Cannot swallow any foods with texture	Flow type: <3 × 10^3^ Gel type: <5 × 10^3^	No identifiable shape; completely smooth, non-solid texture

Flow type and gel type refer to the physical form of the food.

**Table 2:** Comparison of texture classification levels between the IDDSI and UDF systems [7,8,12].

IDDSI Level	UDF	
	Description	Hardness Limit (N/m^2^)
Level 7-Regular	Can be easily chewed	5 × 10^5^
Level 6-Soft & bite-sized	Can be broken up with the teeth	5 × 10^4^
Level 5-Minced & moist	Can be mashed with the tongue	2 × 10^4^
Level 4-Pureed	No need to chew	5 × 10^3^

**Table 3:** Pork main course pureed texture testing.

Ingredient	Dish	IDDSI Level 4			Level 4 Judgement Result	UDF Non-Chewing Grade	Stage 4 Judgement Result	Comparison of IDDSI vs. UDF
		Fork Pressure Test	Fork Drip Test	Spoon Tilt Test		Hardness (N/m^2^)		
Pork leg minced meat	Steamed pork with shiitake mushrooms	Pass	Pass	Pass	**Pass**	274.0 ± 10.0	**Pass**	Consistent
	Minced meat stir-fried with winter melon	Fail	Fail	Fail	**Fail**	92.1 ± 2.6	**Pass**	Inconsistent
	Stir fried pork with holy basil	Pass	Pass	Pass	**Pass**	187.4 ± 6.7	**Pass**	Consistent
Pork shoulder chunks	Pork stew with potato	Fail	Fail	Fail	**Fail**	168.5 ± 2.5	**Pass**	Inconsistent
	Roasted pork with radish	Fail	Fail	Fail	**Fail**	95.1 ± 1.8	**Pass**	Inconsistent
	Steamed pork with glutinous rice flour	Pass	Pass	Fail	**Fail**	293.9 ± 0.6	**Pass**	Inconsistent

UDF non-chewing grade hardness limit was 5 × 10^3^ N/m^2^. Data presents categorical comparisons with IDDSI and UDF (pass/fail only; no statistical analyses applied).

**Table 4:** Chicken main course pureed texture testing.

Ingredient	Dish	IDDSI Level 4			Level 4 Judgement Result	UDF Non-Chewing Grade	Stage 4 Judgement Result	Comparison of IDDSI vs. UDF
		Fork Pressure Test	Fork Drip Test	Spoon Tilt Test		Hardness (N/m^2^)		
Boneless chicken thigh fillet	Boneless chicken thigh fillet with red yeast	Pass	Pass	Fail	**Fail**	389.6 ± 20.2	**Pass**	Inconsistent
	Boneless chicken thigh fillet with salt and pepper	Fail	Fail	Fail	**Fail**	159 ± 1.7	**Pass**	Inconsistent
	Boneless chicken thigh fillet with teriyaki	Pass	Pass	Fail	**Fail**	1004.8 ± 37.8	**Pass**	Inconsistent
Chicken breast	Chicken breast slices stir-fried with eggs	Pass	Pass	Pass	**Pass**	195.8 ± 1.3	**Pass**	Consistent
	Stir-fried chicken breast slices with colorful peppers	Pass	Pass	Pass	**Pass**	204.8 ± 12.8	**Pass**	Consistent
	Stir-fried chicken slices with green papaya	Fail	Fail	Fail	**Fail**	175.1 ± 13.1	**Pass**	Inconsistent

UDF non-chewing grade hardness limit was 5 × 10^3^ N/m^2^. Data presents categorical comparisons with IDDSI and UDF (pass/fail only; no statistical analyses applied).

**Table 5:** Fish main course pureed texture testing.

Ingredient	Dish	IDDSI Level 4			Level 4 Judgement Result	UDF Non-Chewing Grade	Stage 4 Judgement Result	Comparison of IDDSI vs. UDF
		Fork Pressure Test	Fork Drip Test	Spoon Tilt Test		Hardness (N/m^2^)		
Catfish fillet	Stir-fried diced catfish with colorful peppers	Fail	Fail	Fail	Fail	78.8 ± 1.0	Pass	Inconsistent
	Steamed catfish with pesto powder	Pass	Pass	Pass	Pass	321.3 ± 14.1	Pass	Consistent
	Catfish fillet snapper with garlic sauce	Pass	Pass	Pass	Pass	233.2 ± 13.4	Pass	Consistent
Snapper	Grilled snapper with Italian spice powder	Fail	Fail	Fail	Fail	113.6 ± 3.5	Pass	Inconsistent
	Steamed snapper with soy sauce	Fail	Fail	Fail	Fail	95.9 ± 2.5	Pass	Inconsistent
	Grilled snapper with savory crispy beans	Fail	Fail	Fail	Fail	92.7 ± 0.9	Pass	Inconsistent

UDF non-chewing grade hardness limit was 5 × 10^3^ N/m^2^. Data presents categorical comparisons with IDDSI and UDF (pass/fail only; no statistical analyses applied).

**Table 6:** Subjective and instrumental texture evaluation of pureed main dishes after food-shaping agent addition.

Ingredient	Dish	Hardness (N/m^2^)	UDF	IDDSI
Pork leg minced meat	Steamed pork with soft textured shiitake mushrooms	692.9 ± 15.4	Non-chewing grade	Level 4
	Minced meat stir-fried with winter melon	342.4 ± 9.3		
	Stir fried pork with holy basil	456.6 ± 4.1		
Pork shoulder chunks	Pork stew with potato	556.7 ± 10.6	Non-chewing grade	Level 4
	Roasted pork with radish	282.9 ± 7.4		
	Steamed pork with glutinous rice flour	758.6 ± 7.5		
Boneless chicken thigh fillet	Boneless chicken thigh fillet with red yeast	769.1 ± 14.8	Non-chewing grade	Level 4
	Boneless chicken thigh fillet with salt and pepper	1146.8 ± 42.1		
	Boneless chicken thigh fillet with teriyaki	1370.8 ± 69.1		
Chicken breast	Chicken breast slices stir-fried with eggs	748.1 ± 27.9	Non-chewing grade	Level 4
	Stir-fried chicken breast slices with colorful peppers	748.3 ± 16.7		
	Stir-fried chicken slices with green papaya	451.6 ± 19.7		
Catfish fillet	Stir-fried diced catfish with colorful peppers	535.2 ± 19.8	Non-chewing grade	Level 4
	Steamed catfish with pesto powder	804.0 ± 32.9		
	Catfish fillet snapper with garlic sauce	782.1 ± 23.4		
Snapper	Grilled snapper with Italian spice powder	922.0 ± 19.9	Non-chewing grade	Level 4
	Steamed snapper with soy sauce	572.8 ± 24.1		
	Grilled snapper with savory crispy beans	648.0 ± 19.4		

UDF non-chewing grade hardness limit was 5 × 10^3^ N/m^2^. Data presents categorical comparisons with IDDSI and UDF (pass/fail only; no statistical analyses applied).

**Table 7:** Effects of the food-shaping agent on texture parameters of pureed meat dishes (*n* = 18).

All Main Dishes	Before (*n* = 18)	After (*n* = 18)	*p* Value
Hardness (N/m^2^)	231.97 ± 206.66	699.36 ± 261.48	<0.001
Cohesiveness	0.88 ± 0.06	0.78 ± 0.06	0.200
Adhesiveness (J/m^3^)	38.74 ± 56.1	139.21 ± 56.94	<0.001

*p* < 0.05 indicates a statistically significant difference.

**Table 8:** Texture parameters by meat type before and after food-shaping agent addition.

Meat Type	Hardness (N/m^2^)	Cohesiveness	Adhesiveness (J/m^3^)
Before			
Pork	185.16 ± 78.43 ᵃ	0.87 ± 0.04 ᵃ	30.34 ± 28.13 ᵃ
Fish	155.91 ± 90.42 ᵃ	0.88 ± 0.03 ᵃ	12.32 ± 16.85 ᵃ
Chicken	354.84 ± 301.21 ᵇ	0.89 ± 0.08 ᵃ	73.56 ± 79.90 ᵇ
After			
Pork	515.02 ± 173.39 ᵃ	0.77 ± 0.05 ᵃ	99.12 ± 43.70 ᵃ
Fish	710.65 ± 138.78 ᵇ	0.78 ± 0.07 ᵃ	149.78 ± 33.32 ᵇ
Chicken	872.43 ± 302.88 ᵇ	0.78 ± 0.06 ᵃ	168.73 ± 64.15 ᵇ

Data are presented as the mean ± standard deviation based on six independent measurements. Within each time point section (before or after), values in the same column followed by different lowercase letters significantly differ (*p* < 0.05, by the Kruskal–Wallis test with Dunn’s post hoc test).

## Data Availability

The original contributions presented in this study are included in the article. Further inquiries can be directed to the corresponding author.

## Abbreviations

The following abbreviations are used in this manuscript:

IDDSI	International Dysphagia Diet Standardisation Initiative
TPA	Texture Profile Analysis
UDF	Universal Design Foods
TMDs	Texture-Modified Diets
